# Androgen Receptor Regulates Transcription of the ZEB1 Transcription Factor

**DOI:** 10.1155/2011/903918

**Published:** 2011-12-10

**Authors:** Bynthia M. Anose, Michel M. Sanders

**Affiliations:** ^1^Department of Biochemistry, Molecular Biology and Biophysics, University of Minnesota, Minneapolis, MN 55455, USA; ^2^Department of Chemistry, Bethel University, St. Paul, MN 55112, USA

## Abstract

The zinc finger E-box binding protein 1 (ZEB1) transcription factor belongs to a two-member family of zinc-finger homeodomain proteins involved in physiological and pathological events mostly relating to cell migration and epithelial to mesenchymal transitions (EMTs). ZEB1 (also known as *δ*EF1, zfhx1a, TCF8, and Zfhep) plays a key role in regulating such diverse processes as T-cell development, skeletal patterning, reproduction, and cancer cell metastasis. However, the factors that regulate its expression and consequently the signaling pathways in which ZEB1 participates are poorly defined. Because it is induced by estrogen and progesterone and is high in prostate cancer, we investigated whether *tcf8*, which encodes ZEB1, is regulated by androgen. Data herein demonstrate that *tcf8* is induced by dihydrotestosterone (DHT) in the human PC-3/AR prostate cancer cell line and that this induction is mediated by two androgen response elements (AREs). These results demonstrate that ZEB1 is an intermediary in androgen signaling pathways.

## 1. Introduction

ZEB1 (also known in vertebrates as *δ*EF1, zfhx1a, AREB6, TCF8, and Zfhep) and ZEB2 (SIP1) are the closely related human forms of a two-member family of E-box binding transcription factors that is conserved from worm to man (for review, see [1]. Both proteins contain seven zinc fingers, four near the N-terminus and three near the C-terminus, and both can activate [2–4] and repress [5, 6] target genes. ZEB1 is encoded by the *tcf8 *(also sometimes called *zfhx1a *or *zeb1*) gene. Although *tcf8* was cloned in 1991 [7] and the gene that encodes ZEB2 was cloned in 1999 [8], our understanding of the roles of the resultant proteins in normal and abnormal physiology is still emerging. Both appear to promote cell migration during development and during cancer progression [9–14], primarily by repressing the expression of the E-cadherin gene in epithelial cells [1, 9, 10, 15, 16]. However, ZEB1 acts as a tumor suppressor in adult T-cell leukemia/lymphoma [17]. Interestingly, ZEB1 is a key repressor of the *p73* and *p63* genes, members of the *p53* gene family, and its binding to those genes promotes cell proliferation and prevents differentiation [18]. ZEB1 expression is limited in some contexts to proliferating cells by the Rb-E2F1 complex [19]. ZEB1 also opposes fat accumulation in female mice [20] and prevents uterine contraction during pregnancy [21], linking its actions to complex physiological events that are not related to cell migration or EMT.

Despite ZEB1's importance in modulating development, cell proliferation, reproduction, and metabolism, relatively little is known about the regulation of *tcf8. *The actual promoter has only been defined in chicken [22] although promoter-reporter assays suggest that a comparable basal promoter region exists in humans 36 bp upstream of the translation start site [23]. A second more distal promoter may function in some tissues or contexts [24]. The gene is also modulated by a number of signal transduction pathways although the mechanisms remain undefined (for review, see [24]. While growth factors such as the TGF-*β* family [25] and IGF-1 [26] regulate *tcf8*, steroid hormones are major inducers. Notably, expression of ZEB1 is induced by estrogen within one hour in chick oviduct [2]. It is also regulated by estrogen in mouse pituitary [27, 28], in human myometrial cells [12], in mouse adipose tissue (Saykally, Sandri, and Sanders, manuscript in revision), and in human penile tissue and foreskin [29]. This was shown to be at the transcriptional level [2], suggesting that *tcf8 *is a direct target for the estrogen receptor, although no estrogen response element(s) has been identified as yet. Interestingly, estrogen represses expression of ZEB1 mRNA in L*β*T2 gonadotrope cells [28]. ZEB1 mRNA is also induced by progesterone in the human T47D breast cancer cell line [30], in human myometrial cells [12], and in mouse uterus [21], indicating that expression of ZEB1 is steroid-regulated in a variety of tissues.

The goal of these studies was to assess whether androgen receptor (AR) directly regulates *tcf8*. This question is particularly relevant as ZEB1 expression is high in aggressive prostate cancer (PCa) cell lines and tissues and as it promotes phenotypic changes consistent with epithelial to mesenchymal transitions (EMT) in those lines [26, 31]. This raises the possibility that ZEB1 mediates at least some of the effects of AR in promoting PCa progression. Our preliminary results showing that ZEB1 mRNA levels increase in response to androgen treatment in the 22RV1 and PC3/AR prostate cancer cell lines have been published [32, 33]. This study extends those observations to show that androgen affects the transcriptional activity of *tcf8 *through binding or tethering of AR to this gene. Treatment of the PC-3/AR prostate cancer cell line, which overexpresses AR [34], with dihydrotestosterone (DHT) induced endogenous ZEB1 mRNA and protein, confirming our observations that androgen increases ZEB1 mRNA and showing for the first time that this is reflected in ZEB1 protein levels. Inspection of the proximal ~1000 bp of 5′-flanking DNA revealed two potential androgen response elements (AREs). Characterization of these sites revealed that both are required to confer androgen responsiveness to *tcf8* and that both bind AR. This raises the possibility that ZEB1 serves as a direct intermediary in at least some androgen signal transduction pathways.

## 2. Materials and Methods

### 2.1. Cell Culture, Transient DNA Transfections, and *β*-Galactosidase Assays

The PC-3/AR prostate carcinoma cell line was kindly provided by Dr. K. Burnstein (University of Miami) and was maintained as previously described [34]. The LNCaP, C4, C4-2, and C4-2B cell lines were purchased from ViroMed Laboratories (Minnetonka, MN) and were maintained in T-medium (Invitrogen, Carlsbad, Calif, USA) with 10% FBS. Dihydrotestosterone (DHT, D-7149 from Sigma Chemical Co.) dissolved in 95% ethanol was added as indicated. Transient transfection assays were performed in PC-3/AR cells using Lipofectamine with the Lipofectamine 2000 protocol (Invitrogen). Transfections were done on the LNCaP lines using ExpressFect (Denville Scientific, Metuchen, NJ, USA). *β*-galactosidase assays were performed according to the manufacturer's instructions using the Galacto-*Star* System (Applied Biosystems, Foster City, Calif, USA), with chemiluminescence measured in a standard luminometer at 1.0 second/tube.

### 2.2. Real-Time PCR (qPCR)

**Figure 1:**
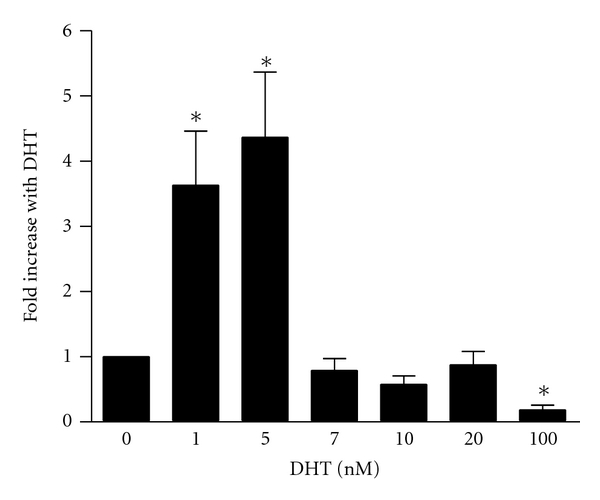
*Endogenous ZEB1 mRNA expression is induced by DHT*. PC-3/AR cells were treated with vehicle (0 nM) or increasing amounts of DHT as indicated. Twenty-four hours later, RNA was harvested and subjected to qPCR using the ZEB1 and RPL32 primers listed in Table 1. ZEB1 mRNA levels were normalized to RPL32 and then plotted relative to the no DHT control. This experiment was repeated 6 times in triplicate. The errors bars represent the standard deviation from the mean average of all experiments. *P < 0.05 compared to no DHT.

Total RNA from cultured cell lines was isolated using TRIzol Reagent (Invitrogen) according to the manufacturer's protocol. Two *μ*g aliquots of mRNA were reverse transcribed with MMLV Reverse Transcriptase (Invitrogen) to create cDNA templates for real-time reverse transcriptase PCR (qPCR). For Figure 1, ZEB1 and RPL32 mRNA were measured simultaneously using TaqMan qPCR in 96-well plates on an *iCycler* (BioRad, Hercules, Calif, USA) using the probes and primers designated in Table 1. Each primer was dual labeled with a fluorescent dye at its 5′ end and a fluorescent quencher molecule at its 3′ end. The emission spectrum for each dye is different, which allows the discrimination of both probes in the same reaction. For ZEB1, the fluorophore was 6-carboxy-4,7,2′,7′-tetrachlorofluorescein (TET) and the quencher was 6-carboxy-*N*, *N*, *N*′, *N*′-tetramethylrhodamine (TAMRA). For *RPL32,* the fluorophore was 6-carboxyfluorescein (6-FAM) and the quencher was TAMRA. Both probes and primer sets were used in each reaction with the following optimized multiplex PCR protocol: 12.5 *μ*L iQ Supermix (BioRad), 2.5 *μ*L 10 mM dNTPs, 2 *μ*L 50 mM MgCl_2_, 0.5 *μ*L iTaq (BioRad), and 0.5 *μ*L cDNA template. Cycling parameters were as follows: 1 cycle at 95°C for 3 minutes, followed by 60 cycles at 95°C for 10 seconds and 55°C for 1 minute.

In Figure 7(a), SYBR Green qPCR was done to quantitate the ZEB1 and GAPDH mRNA using the primers in Table 1 in reactions as follows: 12.5 *μ*L iQ SYBR Green Supermix (BioRad, cat #1708885), 2 *μ*L primer mix (10 *μ*M each), 2 *μ*L template (100 ng/*μ*L), and 8.5 *μ*L H_2_O. This reaction was prepared as a 3x cocktail and distributed in triplicate into a 96-well plate. qPCR was performed using the BioRad iCycler with an annealing temperature of 61°C for both cDNAs.

### 2.3. Western Blotting

Protein extracts were harvested from PC-3/AR cells using the ReadyPrep Protein Extraction Kit (BioRad) according to the manufacturer's protocol. Protein concentrations were determined by Bradford assay (BioRad), and 100 mg of nuclear extract was loaded per well on a 4–20% ReadyGel Tris-HCl (BioRad). Proteins were run at 60 mAmp for 45 min and transferred to a polyvinyldifluoride (PVDF) membrane for blotting with a ZEB1 antibody and a control *α*-tubulin antibody (Santa Cruz Biotechnology, Santa Cruz, CA, E-20 and H-300, resp.) followed by horseradish peroxidase-conjugated secondary antibody (Sigma-Aldrich, St. Louis, Mo, USA). Blots were developed with NBT/BCIP solution (Pierce, Rockford, Ill, USA).

### 2.4. Cloning the 5′-Flanking Region and Proximal Promoter of Tcf8

A 974 bp region of DNA spanning −982 to −9 relative to the translation start site of *tcf8* was cloned by PCR from human genomic DNA (Clontech, Palo Alto, CA) using the forward primer 5′-TGGCCTGTGGATACCTTAGC-3′ (−982 to −963) and reverse primer 5′-CGCTTGTGTCTAAATGCTCG-3′ (−9 to −28) into the pBlueTOPO *β*-galactosidase reporter vector (Invitrogen), and the insert was sequenced. This *tcf8 *promoter construct was named pBlueZEB974.

### 2.5. Plasmid Mutagenesis

Site-directed mutagenesis was carried out on pBlueZEB974 using the QuikChange Mutagenesis kit (Stratagene), following the manufacturer's suggested protocol with the oligomers listed in Table 1 and their corresponding complimentary oligomers. To create the double-mutation, the downstream ARE was initially mutated, and this construct was then used as the template for another round of mutation using the upstream ARE primers. All plasmids were sequenced.

### 2.6. Gel Mobility Shift Assays (GMSAs)

Nuclear protein extracts were harvested from PC-3/AR cells treated with 5 nM DHT using the ReadyPrep Protein Extraction Kit (BioRad) according to the manufacturer's protocol. Protein concentrations were determined by Bradford Assay (BioRad). GMSAs were performed as described [2] using 10 *μ*g of nuclear protein and 80,000 cpm ^32^P-end-labeled oligomer as indicated per lane. The sequences of the top strands are listed in Table 1 with the ARE half-sites underlined.

Antibody supershift reactions were performed using a 0.1 mg/mL final concentration of *α*-AR (A2281x, U.S. Biological, Swampscott, Mass, USA). The immunogen was a synthetic peptide corresponding to residues 299–315 of human AR. GMSAs were placed against film for 48 h at −80°C prior to autoradiographic development.

## 3. Results

### 3.1. Androgen Induces the Expression of ZEB1 MRNA

Nuclear run on [2] and reporter assays [21] indicate that *tcf8 *is induced at the transcriptional level by both estrogen and progesterone, respectively. Because progesterone receptor and AR recognize many of the same binding sites (for recent update, see [35], it seemed plausible that *tcf8* might be a target gene for AR, especially as androgen induces expression of ZEB1 mRNA [32, 33]. To extend these observations, the response of endogenous *tcf8* to dihydrotestosterone (DHT), an androgen that cannot be aromatized to estrogen, was examined. Human PC-3/AR cells were treated with doses of DHT from 1 to 100 nM for 24 hours (Figure 1). Total RNA was harvested and subjected to real-time TaqMan PCR (qPCR) for expression of ZEB1 mRNA using the 60S Ribosomal Protein L32 (RPL32) housekeeping gene [36] as a control. As expected, ZEB1 mRNA was induced about 4-fold by low doses of DHT but did not respond to doses over 7 nM and was significantly repressed by 100 nM DHT. Nonetheless, these data confirm our data in 22RV1 prostate cancer cells [32] that endogenous ZEB1 mRNA levels are increased by androgen.

### 3.2. ZEB1 Protein Also Increases in Response to Androgen Treatment

**Figure 2:**
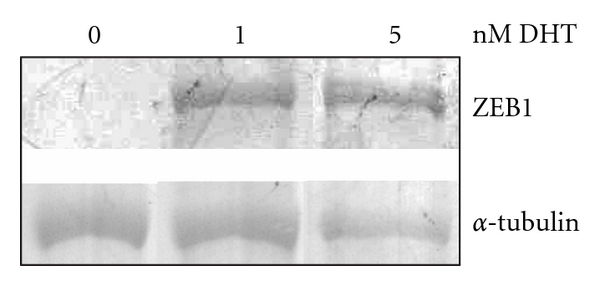
*Expression of ZEB1 protein is induced by DHT*. Protein was isolated from PC-3/AR cells treated with the indicated amounts of DHT for 24 hrs. One hundred mg of protein was used for western blot analysis with a ZEB1 antibody or an *α*-tubulin antibody as a loading control. The ~150 kDa ZEB protein and the 45 kDa *α*-tubulin protein are indicated.

To ascertain whether the increase in ZEB1 mRNA was mirrored by an increase in protein, western blots were performed (Figure 2). These blots revealed that ZEB1 levels do indeed increase from essentially undetectable in response to DHT treatment. Thus, not only are ZEB1 mRNA levels increased by estrogen and progesterone, but they are also regulated by androgen and this is reflected by an increase in ZEB1. This suggests that ZEB1 plays an important role in androgen signaling although no androgen-responsive ZEB1 target genes have been identified as yet.

### 3.3. Androgen Induces Tcf8 at the Transcriptional Level Through Two AREs

**Figure 3:**
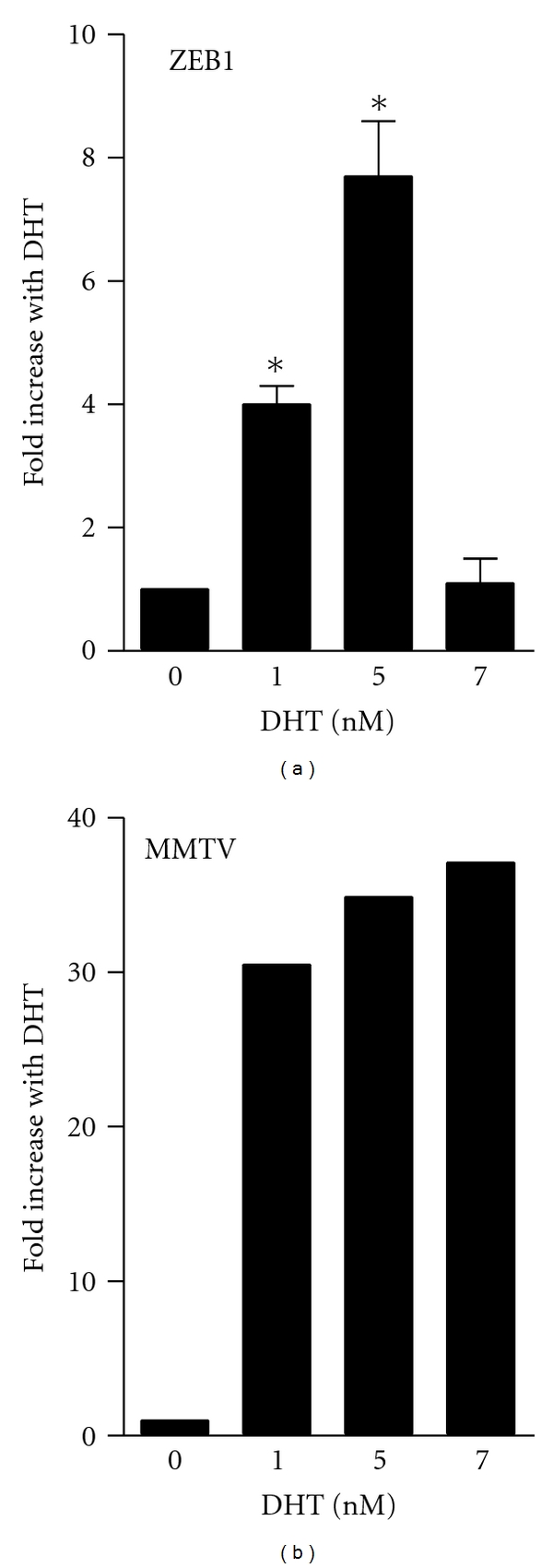
*DHT induces tcf8 at the transcriptional level*. PC-3/AR cells were transiently transfected with a *β*-galactosidase reporter plasmid (pBlueZEB974) containing sequences −982 to −9 from tcf8 (left panel) or the MMTV promoter (right panel) and increasing concentrations of DHT as indicated. *β*-galactosidase activity was determined, and the results are plotted relative to values in the absence of DHT. *n* = 3, *P < 0.5 compared to no DHT.

To ascertain whether *tcf8 *contains any obvious AREs, its sequence was inspected and two putative AREs were identified from −944 to −930 and from −140 to −126 relative to the start site of translation. The start site of transcription has only been experimentally determined in chicken [22] although it is predicted in human to be 36 nucleotides upstream of the translation start site [23]. To investigate whether these potential AREs are functional, a 974 bp genomic fragment (−982 to −9 relative to the translation start site) was subcloned into a *β*-galactosidase reporter vector (called pBlueZEB974), and this plasmid was tested in transfection assays with increasing amounts of DHT (Figure 3(a)). This promoter construct responded to low doses of  DHT but not to higher doses, confirming the results with the endogenous gene (Figure 1). In contrast, the MMTV promoter was induced by all doses of DHT (Figure 3(b)) including doses up to 100 nM (data not shown), suggesting that lack of induction by concentrations higher than 5 nM is selective for the *tcf8* promoter. Because the fold induction of the *tcf8* promoter construct by DHT equals or exceeds that of the endogenous gene, all of the effects of androgen are likely at the level of transcription. These results also demonstrate that this 974 bp region is sufficient for the induction of *tcf8* by androgen.

**Figure 4:**
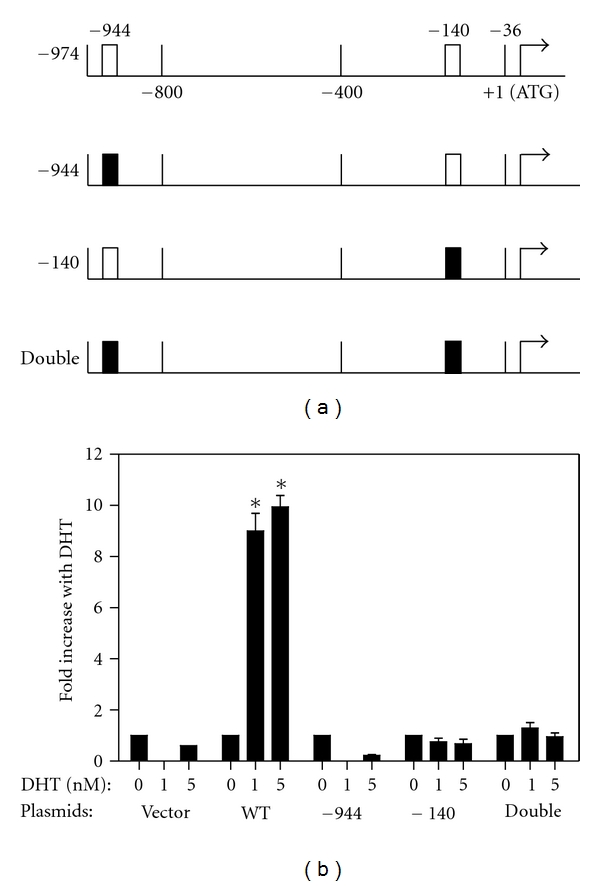
Two functional AREs are present in the *tcf8* 5′-flanking region. (a) Two well-conserved AREs were identified in tcf8 at −944 and −140 (white boxes) relative to the translation start site. Plasmids were constructed in which each of the putative AREs was mutated individually or both were mutated (black boxes). −36 is the putative transcription start site and is numbered relative to the translation start site. (b). The plasmids depicted in (a) were transfected into PC-3/AR cells with increasing concentrations of DHT as indicated. The cells were harvested after 24 hrs and the *β*-galactosidase activity in the lysates was determined. *P < 0.05 relative to no DHT. *n* = 6 with 2-3 replicates per experiment.

In order to determine whether the two putative AREs at −944 and −140 mediate the effects of androgen, point mutations in the critical AR binding residues were introduced into each separately and together (Figure 4(a)). Transient transfections of these plasmids were performed in PC-3/AR cells treated with or without DHT (Figure 4(b)). The data indicate that mutation of either putative ARE completely abolished the responsiveness of *tcf8 *to androgen, confirming that these are in fact authentic AREs.

### 3.4. Androgen Receptor Binds to Both of the AREs

**Figure 5:**
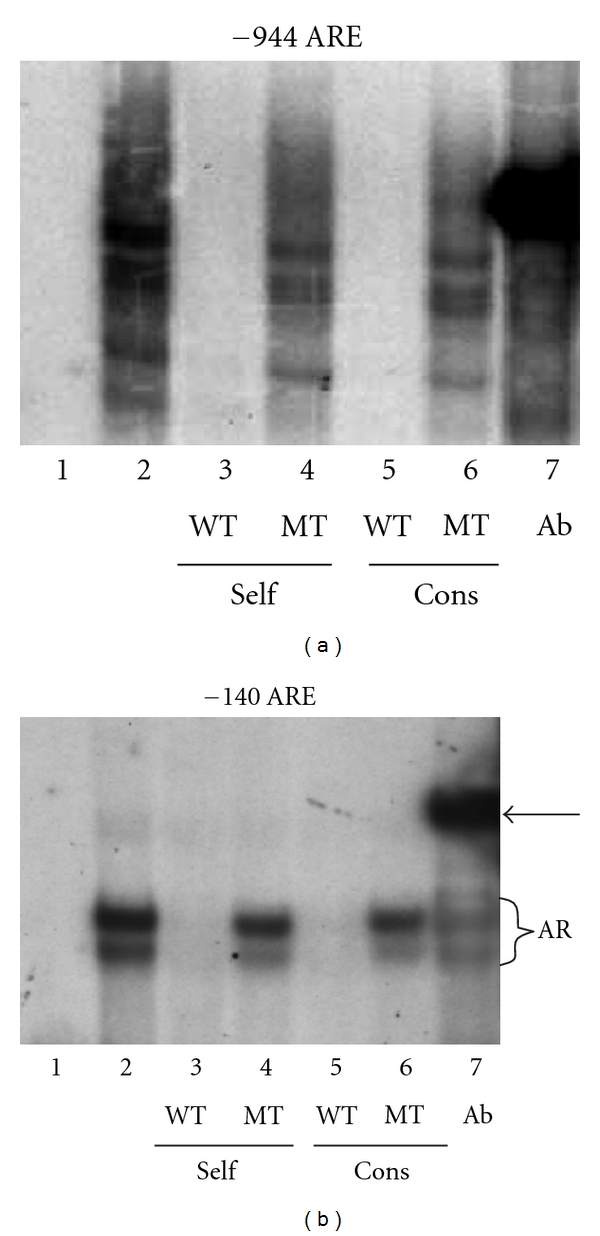
*The AREs in tcf8 bind AR. PC-3/AR cells were treated with 5 nM DHT for 24 hrs, and nuclear protein extracts prepared*. Oligomers corresponding to the regions of DNA containing (a) the −944 ARE and (b) the −140 ARE were radiolabeled and used as probes. Both GMSAs were run in parallel, with only the ARE probe differing. Ten *μ*g of nuclear protein was used in each lane except for Lane 1, which contained only the radioactive oligomer. Lane 2: plus nuclear extract. Lane 3: plus 50x cold wild-type (WT) self-competitor. Lane 4: plus 50x cold self-competitor with a mutated (MT) ARE. Lane 5: plus 50x cold WT consensus ARE. Lane 6: plus 50x cold MT consensus ARE. Lane 7: plus an AR antibody. The arrow indicates the band representing the AR/DNA complexes supershifted by the antibody.

To ascertain whether, as predicted, AR binds to the AREs at −944 and −140, GMSAs were performed using PC-3/AR cell nuclear proteins extracted from cells treated with 5 nM DHT (Figure 5). As is typical with GMSAs using crude nuclear proteins extracts with AREs [37, 38], multiple bands were observed, all of which are competed with wild-type (WT) self-oligomers (lane 3, Figures 5(a) and 5(b)) and an ARE consensus oligomer (lane 5) but not with mutated (MT) self (lane 4) or consensus (lane 6) ARE oligomers. The addition of an AR antibody (lane 7) caused a significant supershift, whereas preimmune serum did not (not shown), verifying that the binding complex for each ARE contains AR.

### 3.5. Endogenous ZEB1 Expression Is Not Induced by Androgen in LNCaP Cell Lines

**Figure 6:**
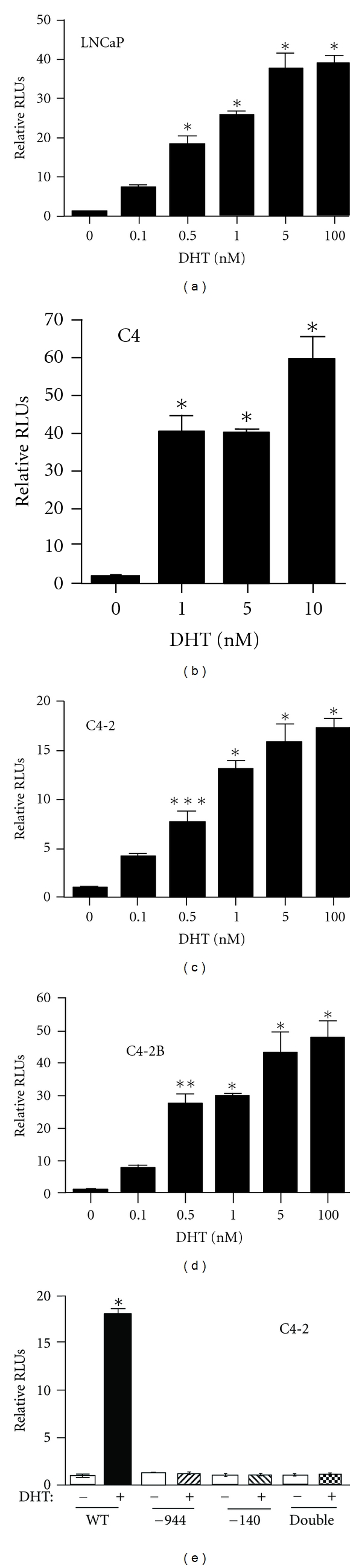
*DHT induces the tcf8 promoter plasmid in a dose-dependent manner in LNCaP cell line derivatives*. The LNCaP (a), C4 (b), C4-2 (c), and C4-2B (d) cell lines were transfected with the pBlueZEB974 promoter plasmid and then cultured with increasing DHT as indicated. The cells were harvested after 24 hrs and the *β*-galactosidase activity in the lysates was determined. (e) C4-2 cells were transfected with the wild type pBlueZEB974 plasmid or with the plasmids containing one or both AREs mutated. Data are plotted relative to the average with no DHT. *P < 0.0001, **P < 0.01, ***P < 0.05.

**Figure 7:**
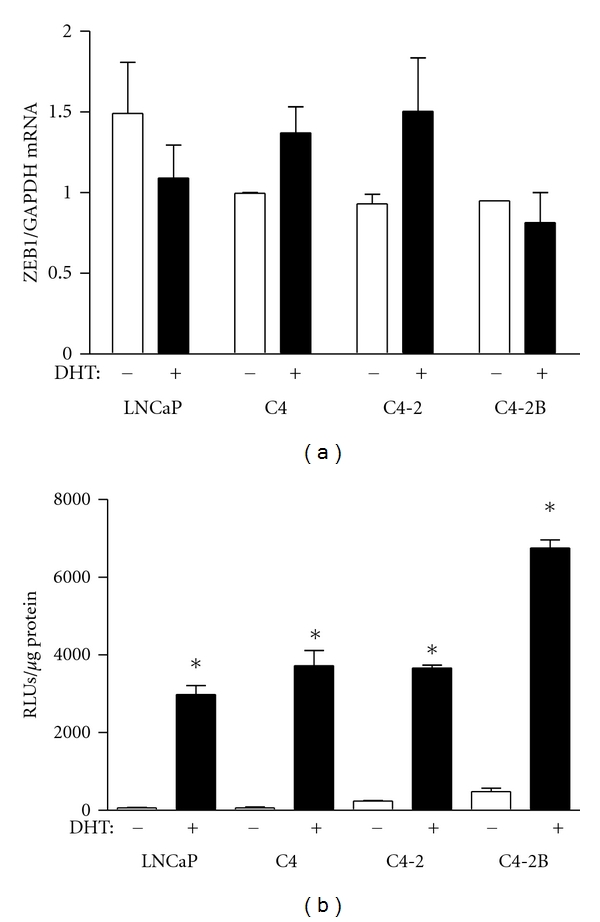
*Endogenous tcf8 is not induced in the LNCaP cell lines*. The four LNCaP-derived cell lines were each divided into two groups and were incubated with or without 5 nM DHT for 24 hrs. (a) For one group, RNA was isolated and subjected to qPCR for ZEB1 and GAPDH mRNA. ZEB1 mRNA levels were normalized to GAPDH. (b) For the second group, the cells were transfected with pBlueZEB974 at T = O, and *β*-galactosidase activity was determined at 24 hrs. *P < 0.0001 compared to no DHT.

The demonstration that *tcf8* is regulated by androgen, the high expression of ZEB1 mRNA in aggressive PCa cell lines, ZEB1's role in PCa cell migration [26, 31], ZEB1's correlation with Gleason grade in human PCa samples [26], and the increased expression of ZEB1 in primary PCa and associated bone metastases [39] raises the possibility that aberrant expression of *tcf8* might promote PCa metastasis. This concept is supported by the observation that expression of ZEB1 becomes estrogen independent in aggressive endometrial and ovarian carcinomas [40]. To examine whether ZEB1 expression becomes independent of androgen during PCa progression, the LNCaP PCa progression model [41] was used (Figures 6 and 7). The LNCaP cell line is an androgen-responsive, prostate-specific antigen (PSA)-secreting, nonmetastatic human prostate cancer cell line established from a lymph node metastasis [42]. The LNCaP C4-2 and C4-2B lines are two derivatives that have metastatic potential. They are androgen independent for growth in castrated hosts but still have androgen-responsive AR. We hypothesized that ZEB1 expression increases independently of androgen in these metastatic lines and contributes to androgen-independent PCa cell proliferation, comparable to what occurs with ZEB1 expression in advanced ovarian and endometrial cancers [40]. If true, this inappropriate expression of ZEB1 might contribute to the androgen insensitivity observed in advanced PCa.

 In order to examine the relative androgen responsiveness of the cell lines, the pBlueZEB974 plasmid was transfected into the LNCaP (Figure 6(a)), C4 (Figure 6(b)), C4-2 (Figure 6(c)), or C4-2B (Figure 6(d)) cell lines with increasing amounts of DHT. In all cases, expression of the *tcf8 *promoter was strongly induced by DHT in a dose-dependent manner, and the level of expression did not correlate with metastatic potential. However, in contrast to what was observed with the PC-3/AR cells (Figure 1), high concentrations also induced expression. As was seen with the PC-3/AR line, both AREs are required for induction of *tcf8 *(Figure 6(e)). Thus, the *tcf8 *promoter is regulated by androgen in LNCaP cell lines and that regulation is dependent upon the two AREs.

In order to determine whether endogenous *tcf8* was induced by DHT in these cell lines and whether it was affected by the metastatic potential of the cells, the four lines were incubated with or without 5 nM DHT, the RNA was isolated, and subjected to qPCR (Figure 7(a)). Surprisingly, *tcf8* was not induced by DHT in any of the lines even though the *tcf8 *promoter construct was in cells from the same experiments (Figure 7(b)). These results were confirmed with other doses of DHT (data not shown). These data indicate that endogenous *tcf8* is differentially regulated by androgen in the PC-3 and LNCaP cell lines even though the isolated promoter is highly induced in both.

## 4. Discussion

Evidence has accumulated from cell lines and human samples to suggest that ZEB1 plays a role in the progression of PCa [14, 26, 31, 39]. As ZEB1 mRNA is induced by both estrogen [2] and progesterone [30], it seemed plausible that it might also be regulated by androgen, especially as AR and PR can bind to the same regulatory element (for review, see [35]). Our preliminary studies revealed that it was indeed regulated by androgen in both PC3/AR and 22Rv1 prostate cancer cell lines [32, 33]. These studies extend those observations and show that DHT induces endogenous ZEB1 mRNA, but over a fairly small range of concentrations in the PC3/AR cells (Figure 1). A similar dose response was seen with a *tcf8 *promoter construct but not with the MMTV promoter (Figure 3). In contrast, this same promoter reporter vector was responsive to a wide range of androgen concentrations in LNCaP cell lines (Figure 6) indicating that this restriction is cell type specific, selective to the *tcf8 *promoter, and not a consequence of posttranscriptional regulation. The reason for the restricted dose responsiveness of *tcf8* to androgen in PC3/AR cells remains to be elucidated. Nonetheless, our data indicate that androgen induces the transcription of *tcf8* 4- to 9-fold in PC-3 cells, and the effect is even greater at the protein level as little basal expression could be detected (Figure 2). Our data also show for the first time that the androgen induces ZEB1 protein as well as the mRNA (Figure 2).

Transient transfection assays with *tcf8 *promoter constructs revealed that both putative AREs (−944 and −140 relative to the translation start site) are functional and are required for induction (Figure 4). As neither of these are perfect AREs and as they are about 800 bp apart, it may be that a looping mechanism brings the two AREs together to provide sufficient affinity to get induction by androgen. A similar mechanism occurs in the PSA promoter, where an upstream enhancer containing an ARE loops to interact with two promoter-proximal AREs at −400 and −170 [43].

These data support the concept that androgen elicits a transcriptional cascade through the induction of *tcf8*. This is also supported by a recent paper showing that AR induces expression of ZEB1 in triple negative breast cancer cells [44]. Furthermore, treatment of these cells with the antiandrogen bicalutamide reduces ZEB1 protein expression although no mechanism was provided. However, it was somewhat surprising that androgen does not induce endogenous *tcf8 *in the androgen-responsive LNCaP cell line derivatives (Figure 7), even though the promoter construct is responsive (Figure 6). A similar conundrum was observed with the induction of *tcf8* by estrogen [40]. Few estrogen-responsive cell lines show estrogen-dependent expression of ZEB1. One explanation may be that epigenetic silencing of the *tcf8* locus occurs in some cell types. This was shown to be the case in adult T-cell leukemia and lymphoma cells, where epigenetic silencing of *tcf8* appears to contribute to malignancy [17]. Another possibility is that the *tcf8 *gene is actually induced by androgen in LNCaP cell lines, but the mRNA is rapidly degraded by microRNAs (miRNAs). Considerable evidence indicates that there is a reciprocal relationship between ZEB1 and several miRNAs, most notably those of the miR-200 family (for review, see [45]. The ZEB1 3′-UTR contains eight seed sequences for the miR-200 family, and expression of ZEB1 mRNA is inversely correlated with expression of the miR-200 family [45]. These seed sequences are not present in the *tcf8* promoter construct. Moreover, LNCaP cells have extremely high levels of miR-200c, about 1,420-fold that of normal prostate fibroblasts [46]. Additional support for this hypothesis comes from the inverse relationship seen between miR-200 and ZEB1 in a different PC3 cell line derivative [47]. Thus, androgen may in fact be inducing ZEB1 mRNA in LNCaP cells, but it is rapidly degraded by miR-200c.

Although ZEB1 is regulated by estrogen, progesterone, and androgen [2, 12, 21, 27, 28], only a couple of publications address its normal role in mammalian reproduction [12, 21]. Spoelstra et al. [12] showed that ZEB1 is expressed in normal virgin and pregnant mouse myometrium and stroma, but not in epithelial cells, even though those cells also contain estrogen and progesterone receptors. This supports our observations that the presence of steroid receptors is not sufficient to induce *tcf8 *in all cell types (this manuscript and [40]). In the pregnant uterus, progesterone induces expression of ZEB1, which inhibits genes associated with uterine contraction [21]. Interestingly, the miR-200 family is induced at term, repressing ZEB1 expression and allowing parturition. Nothing is known about ZEB1's role in the male reproductive tract. One might speculate that ZEB1 mediates some of androgen's signaling during prostate formation. An AR-dependent signal from the urogenital mesenchyme is required during prostate development (for review, see [48]). As ZEB1 expression in normal cells is primarily mesenchymal [12, 40], it may be that ZEB1 mediates androgen's actions during the development of the prostate and in some aspects of normal male reproductive function. The contribution that ZEB1 makes to the physiological and pathological signaling pathways triggered by androgen remains to be established.

In addition to demonstrating that *tcf8* is regulated at the transcriptional level by androgen, the data herein show that it is a direct target for AR and identify its specific binding sites. This is an important contribution as very few regulatory elements have been characterized in *tcf8*, although a number have been proposed (for review, see [24]) including two progesterone response elements that do not correspond to our proposed AREs [21]. In fact, not even the transcription start site has been experimentally defined in mammals. Characterization of the normal regulation of *tcf8* and expression of ZEB1 is essential for understanding its aberrant expression in various carcinomas, including PCa. These data define a previously uncharacterized mechanism for the regulation of *tcf8 *and provide the basis for additional studies defining its function in the male.

## 5. Conclusion

The *tcf8 *gene is a direct target for AR, which binds to two AREs in the 1000 bp proximal to the translation start site. The increase in expression of this gene is reflected by an increase in ZEB1 protein. However, expression of *tcf8 *is silenced in some reproductive cell lines including the LNCaP PCa cell line by mechanisms that remain to be elucidated but that may involve epigenetic silencing or the miR-200 family of microRNAs.

## Figures and Tables

**Table 1:** The oligomer sequences used for qPCR, mutagenesis, and GMSA.

Oligomer	Oligomer Sequence	Use
ZEB1 (F)	5′-TCCTGAGGCACCTGAAGAGG-3′	PCR
ZEB1 (R)	5′-CAGAGAGGTAAAGCGTTTATAGCC-3′	PCR
RPL32 (F)	5′-CCATCTCCTTCTCGGCATCAT G-3′	PCR
RPL32 (R)	5′-GGT TTCCGCCAGTTACGCTTA-3′	PCR
GAPDH (F)	5′- GACCCCTTCATTGACCTCAACTACATG-3′	PCR
GAPDH (R)	5′-CTCCTGGAAGATGGTGATGGG-3′	PCR
MT ARE (−944 to −930)	5′GAGCCTCTAGGTGTgAattcGGTGAgaaCGcAAAGCCGGGAGTGT3'	Mutation
MT ARE (−140 to −126)	5′GAGCCTCTAGGTGTgAattcGGTGAgaaCGcAAAGCCGGGAGTGT3'	Mutation
WT ARE (−944 to −930)	5′-agcttCCTAGGATCCCACGGTTCTACGCGAGGAAGAGa-3′	GMSA
MT ARE (−944 to −930)	5′-agcttCCTAGtcTCCCACGtaTCTACGCGAGtcAGAGa-3′	GMSA
WT ARE (−140 to −126)	5′-agcttTGTAAGGAAGGTGATGTCGTAAAGCCa-3′	GMSA
MT ARE (−140 to−126)	5′-agcttTGTgAattcGGTGAgaaCGcAAAGCCa-3′	GMSA
WT Consensus ARE	5′-agcttCTAGAAGTCTGGTACAGGGTGTTCTTTTTGCAa-3′	GMSA
MT Consensus ARE	5′-agcttCTAGAAGTCTGcaACAGGGTcaTCTTTTTGCAa-3′	GMSA

For the site-directed mutagenesis and GMSA oligomers, only the top strand is shown. Small letters indicate mutations or restriction enzyme sites.
